# Citizen science facilitates first ever genetic detection of wolf‐dog hybridization in Indian savannahs

**DOI:** 10.1002/ece3.10100

**Published:** 2023-05-17

**Authors:** Abhinav Tyagi, Mihir Godbole, Abi Tamim Vanak, Uma Ramakrishnan

**Affiliations:** ^1^ National Centre for Biological Sciences Tata Institute of Fundamental Research Bengaluru Karnataka India; ^2^ SASTRA Deemed to be University Thanjavur Tamilnadu India; ^3^ The Grasslands Trust Pune Maharashtra India; ^4^ Ashoka Trust for Research in Ecology and the Environment Bengaluru Karnataka India; ^5^ School of Life Sciences University of KwaZulu‐Natal Durban South Africa

**Keywords:** *Canis lupus*, conservation genomics, ddRAD, hybridization, non‐invasive samples, SNPs

## Abstract

Human demographic expansion has confined wildlife to fragmented habitats, often in proximity to human‐modified landscapes. Such interfaces facilitate increased interactions between feral or domesticated animals and wildlife, posing a high risk to wild species. This is especially relevant for free‐ranging dogs (*Canis lupus familiaris*) and wild canids like gray wolves (*Canis lupus*) and golden jackals (*Canis aureus*). Wolf–dog hybridization may lead to a significant reduction of specific adaptations in wolves that could result in the decline of wolf populations. Detection and genetic discrimination of hybrids between dogs and wolves are challenging because of their complex demographic history and close ancestry. Citizen scientists identified two phenotypically different‐looking individuals and subsequently collected non‐invasive samples that were used by geneticists to test wolf‐dog hybridization. Genomic data from shed hair samples of suspected hybrid individuals using double‐digest restriction‐site‐associated DNA (ddRAD) sequencing resulted in 698 single nucleotide polymorphism (SNP) markers. We investigated the genetic origin of these two individuals analyzed with genetically known dogs, wolves, and other canid species including jackals and dholes (*Cuon alpinus*). Our results provide the first genetic evidence of one F2 hybrid and the other individual could be a complex hybrid between dogs and wolves. Our results re‐iterate the power of next‐generation sequencing (NGS) for non‐invasive samples as an efficient tool for detecting hybrids. Our results suggest the need for more robust monitoring of wolf populations and highlight the tremendous potential for collaborative approaches between citizens and conservation scientists to detect and monitor threats to biodiversity.

## Graphical abstract

Free‐ranging dogs can severely impact wildlife, especially other canid species through predation, scavenging, disease transmission, and hybridization. Detection and genetic discrimination of hybrids between dogs and gray wolves are challenging due to complex demographic history and close ancestry. In this study, we report the first‐ever detection of a wolf‐dog hybrid using genetic and photographic evidence in peninsular India with the help of citizen science.

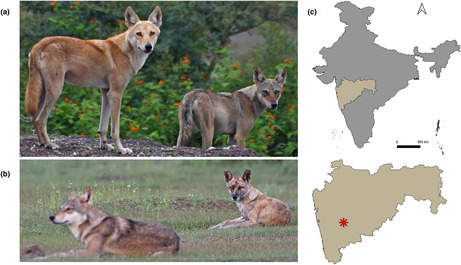

## 1 INTRODUCTION

Increased human‐wildlife interfaces result in escalated interactions between wildlife and domesticated animals (Hindrikson et al., 2012). In particular, free‐ranging dogs (*Canis lupus familiaris*) can severely impact wildlife through direct predation, scavenging, disease transmission, and hybridization (Vanak & Gompper, 2009). Hybridization with subsequent introgression between well‐defined taxonomic entities can pose crucial conservation problems, especially when it involves a domesticated species and wild counterparts (Hindrikson et al., 2012). Such hybridization has been well‐documented and widely studied in various species including both terrestrial and marine taxa (Howard‐McCombe et al., 2021; Pilot et al., 2018; vonHoldt et al., 2013; Wringe et al., 2018).

The gray wolf (*Canis lupus*) is the largest member of the Family *Canidae* and inhabits heterogeneous terrestrial habitats including human‐dominated landscapes (Ripple et al., 2014; Singh & Kumara, 2006). In India, wolves and domestic dogs coexist throughout the wolf's distribution range and share a complex relationship that involves competition and potential hybridization (Home et al., 2018; Srivathsa et al., 2019). The recent availability of genomic resources for both domestic dogs and wolves provides new insights into wolf‐dog hybridization (Pilot et al., 2018). Studies based on genome‐wide analysis demonstrate that wolf populations have retained their genetic diversity and integrity despite recurrent hybridization with dogs (Pilot et al., 2018). However, the increased frequency of hybridization with dogs could pose a serious threat to wild wolf populations (Hindrikson et al., 2017). Wolf–dog hybridization and introgression of dog genomes could lead to the loss of unique and adaptive variation in wolves, posing a significant conservation challenge (Randi, 2008; Salvatori et al., 2020). Besides hybridization, wolf populations risk exposure to rabies, canine distemper, and canine parvovirus disease, which are prevalent in dogs (Thompson et al., 2020).

Identification of hybridization events between wild species and their domestic counterparts is not trivial due to the complex history and genetic ancestry of both subspecies (Stronen, Aspi, et al., 2022; Stronen, Mattucci, et al., 2022). Advances in conservation genomics and increased use of single nucleotide polymorphism (SNP) markers hold promise with higher efficacy in the identification of such events over traditionally used mitochondrial DNA and microsatellite markers (Harmoinen et al., 2021; Stronen, Mattucci, et al., 2022). Poor local capacity to adapt and implement these techniques, especially in developing countries with high biodiversity, often results in a lack of attention to such critical conservation problems (Khan & Tyagi, 2021).

In India, prioritizing sites to investigate wolf–dog hybridization is difficult because (1) the country is large and megadiverse, and (2) the scale and breadth of the human–wildlife interface is substantive. Moreover, wolves mostly occupy savanna grassland and scrub habitats outside designated protected areas, beyond the jurisdiction of the state forest departments. In such scenarios, involving citizen scientists can offer innovative and effective avenues for monitoring wildlife populations and habitats that require conservation interventions (Granroth‐Wilding et al., 2017). India has a rapidly expanding culture of citizen scientists with an interest in nature and wildlife, promoting an environment that can result in the co‐production of knowledge for conservation (SoIB, 2020; ebird; *ebird.org/india/*).

Despite its immense potential, citizen science is yet to be widely and effectively used for wildlife monitoring across the tropics or the global south countries. There have been very few studies and initiatives in India, which have combined the efforts of citizens and scientists in wildlife conservation studies. Here we document a case of wolf–dog hybridization in India along with two lines of evidence. Morphological characteristics alone might not be sufficient to identify hybrids (Galaverni et al., 2017). In this paper, we used both photographic and genetic evidence together to identify hybrids. To the best of our knowledge, this is the first evidence‐based documentation of wolf–dog hybridization in the country and also the first instance where citizens and scientists have collaborated for such a discovery. Our findings point to the possibility of setting up robust and well‐organized citizen science programs for monitoring wild wolf populations and tracking wolf–dog hybridization events in the future.

## 2 METHODS

### 2.1 Sample collection and library preparation

**FIGURE 1:**
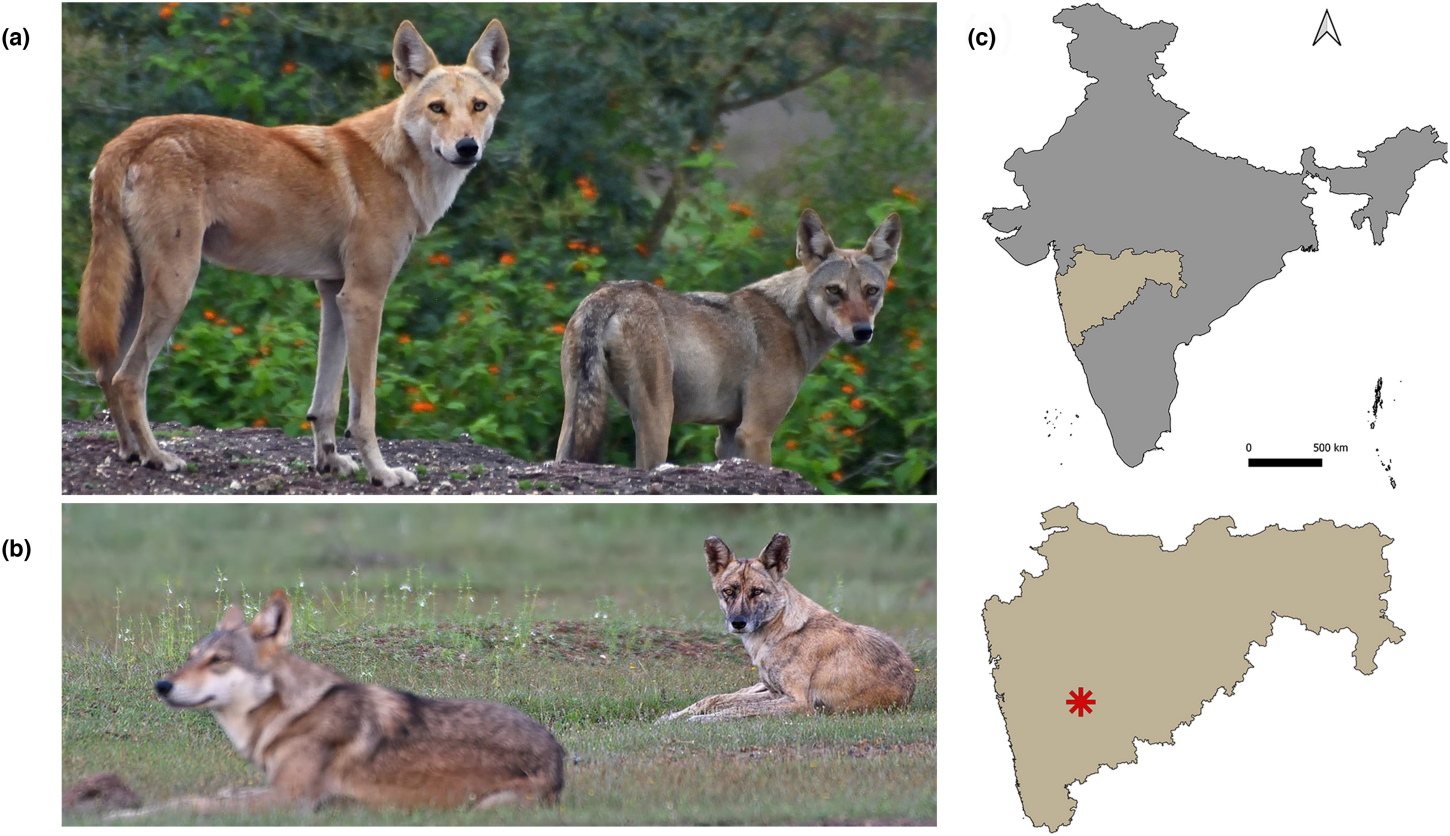
Photographic capture of the suspected wolf‐dog hybrid individuals. (a) H1 (with a tawny coat) along with a wolf in the background, (b) H2 (with unusual facial features) with a wolf in the foreground, spotted near Pune city (shown as a red star on the map) in Maharashtra, India (c). (Photo credit: Siddhesh Bramhankar).

A suspected wolf–dog hybrid individual with an unusual tawny coat was first sighted and photographed by a group of nature enthusiasts near Pune city in western India (Figure 1a). The observers documented two such instances where an individual in the wolf pack looked different from the others (Figure 1a,b). Trailing these suspected hybrid individuals, shed hair samples were non‐invasively collected from resting sites. Each sample consisted of 8–10 hair strands and was stored in zip lock bags before transport to the laboratory at room temperature.

We extracted DNA from the hair samples along with two extraction controls, using DNeasy Blood & Tissue extraction kit (Qiagen). Prior to extraction hair samples were rinsed with 0.1X commercial bleach, washed with ethanol, and nuclease‐free water in order to remove any DNA on the surface (Khan et al., 2020). Double‐digest restriction‐site‐associated DNA (ddRAD) libraries were constructed for both samples post‐concentrating using speedvac (labconco), following the protocol used in Tyagi et al. (2022). Restriction enzymes *Eco*RI and *Sph*I were used to digest the DNA followed by adapter ligation, indexing, and size selection. Final libraries were sequenced at 2 × 100 pair‐end on the Illumina HiSeq2500 platform.

Raw reads were demultiplexed and mapped to the dog reference genome downloaded from the NCBI database (Dog10K_Boxer_Tasha; RefSeq accession: GCF_000002285.5) using BWA mem (Li, 2013). These samples were analyzed along with available whole genome sequences of village dogs and wolves. We used 11 wolf whole genomes including three from North America and Europe, three from west and central Asia, and five from Indian wolves (four are from the same region as suspected hybrids, Hennelly et al., 2021). Sixteen dog genomes were used for the analyses, including dogs from India, China, Nepal, East Asia, and Kenya (Auton et al., 2013; Pendleton et al., 2018). We also investigated the possibility of admixture with other canid species by using three genomes each of golden Jackal and dhole. Accession numbers and details of all 33 genomes used can be found in Table S1.

### 2.2 Variant calling and analysis

Freebayes (Garrison & Marth, 2012) was used for variant calling, and positions supported by at least three reads were considered to identify genotypes, and only biallelic variants (SNPs) were retained. VCFtools (Danecek et al., 2011) was used to filter sites based on site quality (≥20), genotyping quality (≥20), and minor allele count (≥3). Minor allele count (mac) filter was used subsequently after removing individuals (dholes, jackals, and non‐Indian wolves respectively).

To test for hybridization, we used three approaches (Harmoinen et al., 2021). First, we conducted a principal component analysis (PCA) using PLINK (Purcell et al., 2007). Second, we used model‐oriented approaches to determine admixture probabilities using the programs ADMIXTURE (Alexander et al., 2009) and STRUCTURE (Pritchard et al., 2000). We ran Admixture for *K* ranging from one to eight for 10 replicates each, the best *K* was determined by estimating the cross‐validation error. We ran Structure for 1,000,000 MCMC iterations after 100,000 burn‐in iterations with 10 replicates for *K* from one to six. We used the correlated allele frequencies and admixture model. We also calculated the 90% probability intervals, to evaluate membership in the observed clusters. The best *K* was selected by likelihood values and method described by Evanno et al. (2005). The results were analyzed and plotted using a web‐based server, CLUMPAK (Kopelman et al., 2015). Finally, we used a model‐based program NEWHYBRIDS (Anderson & Thompson, 2002) with and without prior information about parental individuals (i.e., z‐option). We ran the program for an initial burn‐in of 10,000 iterations followed by 50,000 iterations.

## 3 RESULTS

We successfully extracted DNA from hair samples of both individuals (referred to as H1 and H2 from hereon) collected by the citizen scientists, and followed it up with constructing and sequencing ddRAD libraries. The number of reads obtained and mapped to the dog reference genome varied slightly between both the samples (7,144,405 and 7,026,501 for H1 and H2 respectively).

**FIGURE 2:**
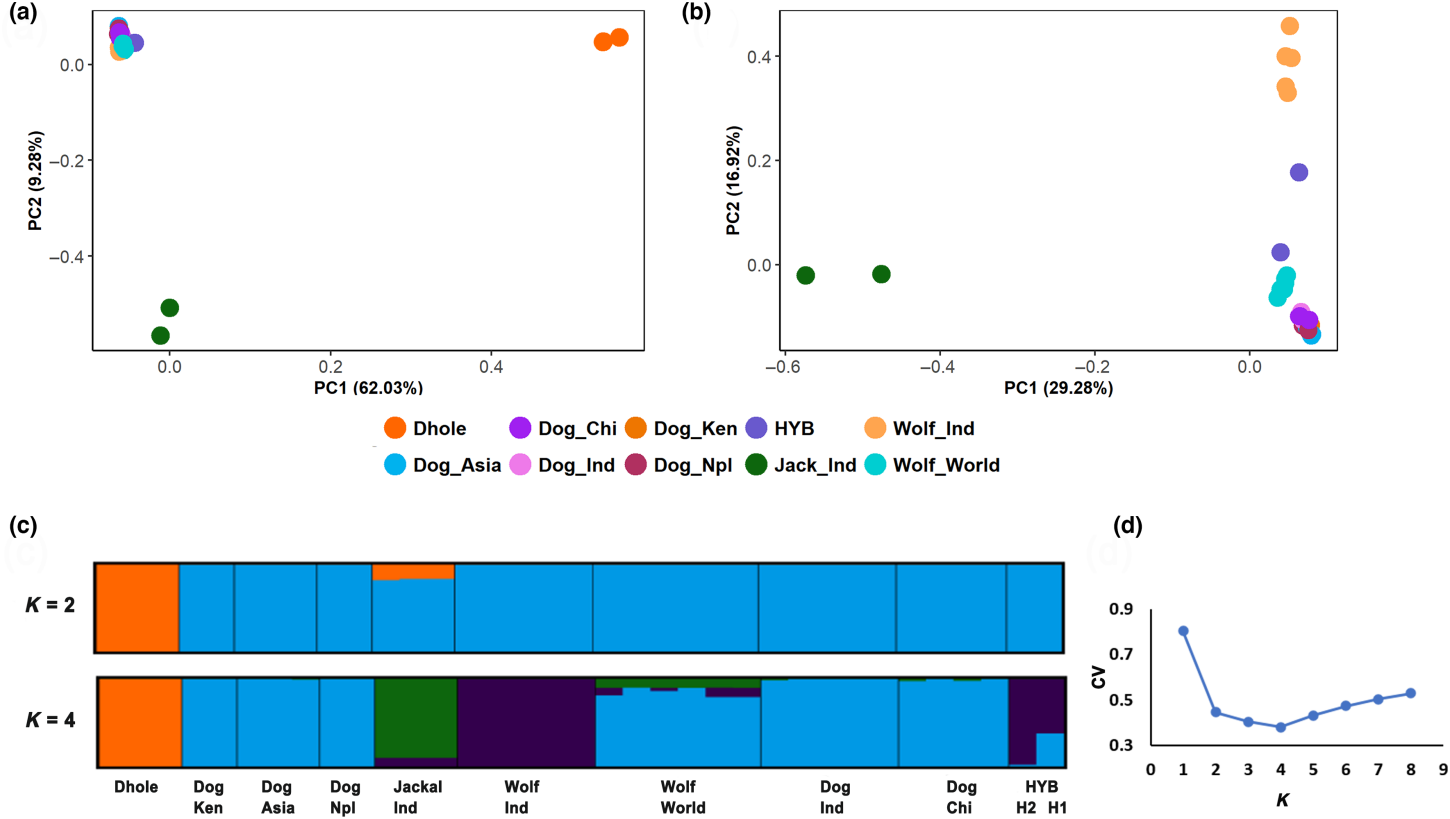
(a) Principal component analysis (PCA) based on 1573 SNPs using all 35 samples, showing clustering of dhole, golden jackal, dogs, and wolves. (b) PCA post removing dhole samples, shows the segregation of jackals at PC1 with the rest of the samples and the separation of Indian wolves, suspected hybrids, wolves from other geographic locations, and dogs at PC2. (c) Admixture plot using all 35 samples, results for *K* = 2 and 4. Each vertical line represents a unique individual. Samples are color‐coded based on species identity. (d) Estimation of cross‐validation error with the number of clusters (*K*) ranging from 1 to 8.

**FIGURE 3:**
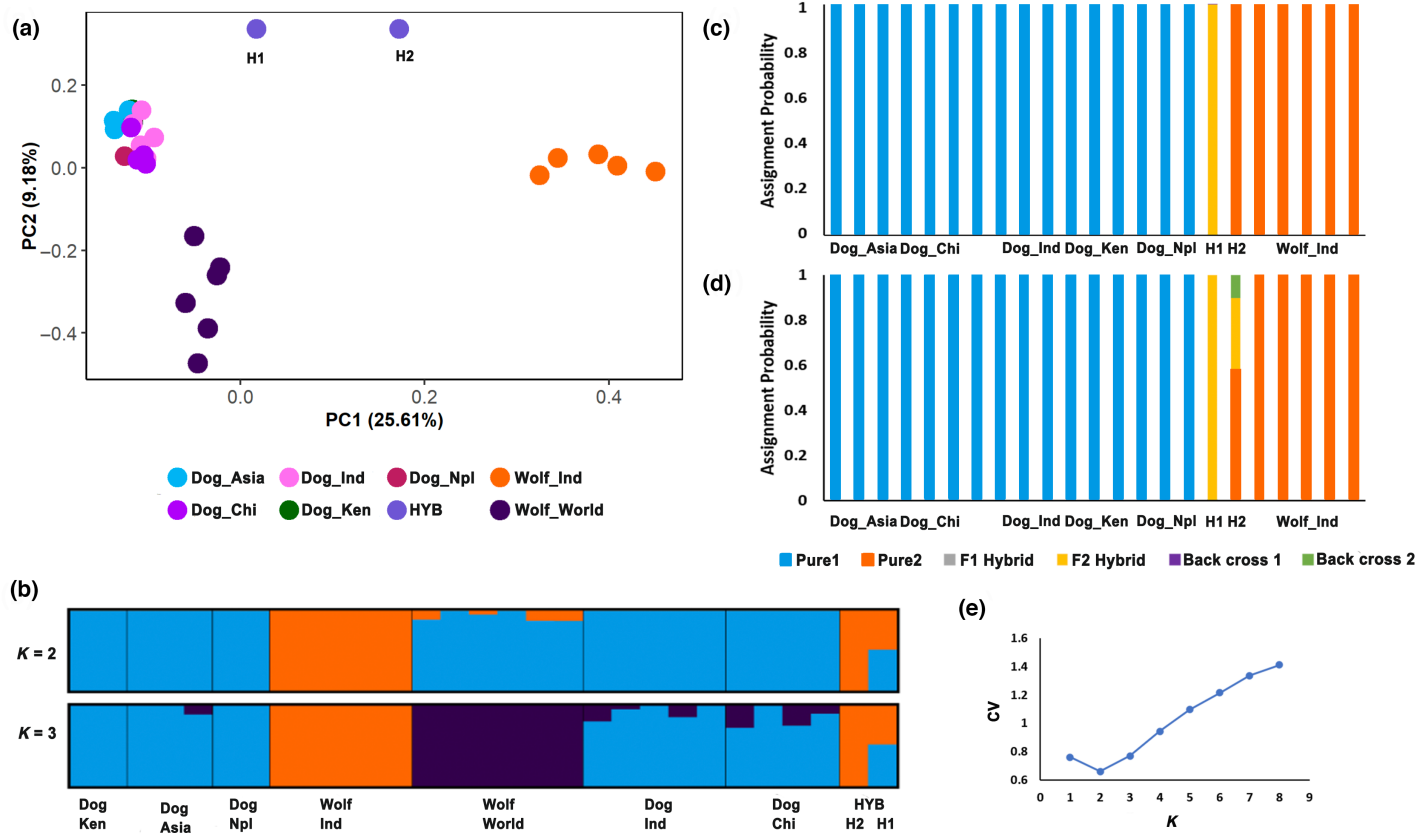
(a) Principal component analysis (PCA) based on 698 SNPs showing clustering of Indian wolves, rest of the world wolves, suspected hybrid individuals, and dogs at PC1 & PC2 (b). Admixture plot results for *K* = 2 and 3. Each vertical line represents a unique individual. Samples are color‐coded based on species identity. The best‐supported *k* value was *K* = 2 (e). (c) Plot showing assignment probabilities from Newhybrids analysis without using any prior information. H1 was assigned as the F2 hybrid category and H2 was assigned with wolves. (d) Assignment probabilities were obtained using prior information about all wolf samples (using the ‐z option). H1 was still assigned to F2 hybrid category but H2 showed a complex mixture of pure wolf, F2, and backcross to wolves.

The first set of filtering using all 35 samples resulted in 1573 SNPs. These were used to perform PCA and Admixture to identify the origin of H1 and H2, with several canid species. We observed clear segregation of Dhole, Jackal, and the wolf‐dog cluster (Figure 2a,b). To better understand wolf‐dog dynamics, dhole, and jackal samples were removed. After applying the appropriate mac filter, the dataset consists of 29 samples and 698 SNPs. PCA analysis showed substantial differentiation between dogs, Indian wolves, and wolves from rest of the world (North America, Europe, West & Central Asia); both suspected hybrid samples were placed in between the Indian wolf and dogs cluster (Figure 3a).

Results from Structure and Admixture were consistent for *K* values between two and four and supported the observations of PCA analysis. Using all 35 individuals, four clusters were identified by both methods (best support for *K* = 4). The clusters included dhole, jackal, Indian wolves, and rest of the world wolves (Figure 2c,d; Figure S1).

The smaller dataset with only wolves and dogs also resulted in consistent Structure and Admixture results for *K* = 2 and *K* = 3. At *K* = 2, Indian wolves were distinguished from dogs and other wolves, and at *K* = 3, dogs, Indian wolves, and wolves were identified as three distinct groups (Figure 3a). Results from both methods were not consistent regarding the optimum *K* value. For Admixture, the lowest cross‐validation error was obtained for *K* = 2 (Figure 3e). Whereas, for Structure, *K* = 3 was the optimum *K* (Figure S2a). However, our inference about the suspected hybrid individuals was not affected by these patterns. Both suspected individuals clustered with wolves at *K* = 2, but the individual H1 had about 42% assignment to the dog cluster in both Structure and Admixture, suggesting that it could be an F2 hybrid.

For NEWHYBRIDS analysis we removed wolves from other populations and only retained five Indian wolves. After applying mac filter post‐sample removal, the final data set consists of 23 individuals and 601 SNPs which were used for the analysis. We ran NEWHYBRIDS twice with and without prior information on parental or pure samples. Without specifying prior information, all wolf, and dog samples were categorized correctly (Figure 3c). One of the two suspected hybrids (H1) was classified as an F2 hybrid, while H2 was classified as a wolf (Figure 3c). After providing prior information about known wolf samples as parental (using z‐option), H1 was still assigned as hybrid (F2), but interestingly, H2 was assigned a complex genetic ancestry, a mixture of the wolf, F2, and a back cross to wolf (Figure 3d). In summary, our results confidently assigned one sample to the hybrid category. However, our results were not consistent for individual H2. This could be due to insufficient data or complex hybridization, which our analyses were unable to identify explicitly.

## 4 DISCUSSION

Our genomic results document the occurrence of wolf‐dog hybridization in peninsular India and, along with photographs of individuals with unusual phenotypes, highlight the complexity, and extent of current hybridization and dog introgression into the wolf population. Although wolf–dog hybridization has long been speculated to be prevalent in India, there are no published reports (to the best of our knowledge) with confirmatory genetic analysis. However, mating events between dogs and wolves have been detected (Hennelly et al., 2015). Such lack of confirmation could be due to relatively few laboratories that integrate field sampling with conservation genomics in the country (Khan & Tyagi, 2021). Alternatively, it is also likely that such hybridization events have low detection rates.

Hybridization among canid species is complex. Both sexes of canid hybrids are fertile (Mech et al., 2017), in contrast to the other mammalian species where males are sterile with very few exceptions (Genualdo et al., 2021). This makes the introgression of dog genome into wolves and vice versa possible. Additionally, high population turnover and loss of breeding members may cause the break‐up of wolf packs and disruption of social structure, which can also further increase hybridization rates (Randi et al., 2014; Stronen, Aspi, et al., 2022). Such scenarios might lead these wild populations into a ‘hybridization vortex’ and subsequently toward ‘extinction via hybridization’ (Stronen, Aspi, et al., 2022). Most conservation efforts in India are focused on issues like examining connectivity (Thatte et al., 2021), tracking population dynamics, monitoring and assessing prey base (Qureshi et al., 2023), and understanding human‐wildlife conflict (Srivathsa et al., 2019). However, for certain species like wolves, which inhabit regions beyond protected areas and face substantial human‐dog interaction, the issue of hybridization is of significant importance for conservation but remains neglected. We suggest that conservation and management tool kits should specifically include hybridization assessment in the future.

Most monitoring and reporting of such events in the country is done by either camera traps to monitor large carnivores like tigers or by the observation of tourists and wildlife enthusiasts. In India, wildlife tourism is a huge and growing avenue that has both positive and negative impacts on wildlife (Tyagi et al., 2019).

Morphological identification of early‐order hybrids may be relatively easy because of their uncharacteristic or atypical physical appearance. However, complex or higher‐order of hybrids might not be morphologically distinct and therefore harder to detect in the wild. Here, we demonstrate how “eyes on the ground” through citizen groups can help investigate rare hybridization events. Systematic citizen science programs have shown great success in using data collected by the public to understand species distribution and dynamics (e.g., eBird). But very few studies have explored monitoring of wildlife populations through a combination of citizen science and non‐invasive sampling (Granroth‐Wilding et al., 2017). Additionally, several issues like data quality and integrity could affect inferences based on citizen science information (McKinley et al., 2017). However, with proper training and planning, complex questions across broad geographic scales can be addressed using data generated through citizen science. Such approaches to biodiversity monitoring can have long‐term benefits to conservation (MacPhail & Colla, 2020).

Most of the information on wolf–dog hybridization comes from extensive studies in European countries based on invasive and non‐invasive samples (Harmoinen et al., 2021; Pilot et al., 2018; vonHoldt et al., 2013). Few studies exist from other parts of the world (Khosravi et al., 2015; Kopaliani et al., 2014; Mallil et al., 2020). Several of these studies have emphasized using a higher number of markers or ancestry‐specific marker panels to identify and monitor hybridization (Harmoinen et al., 2021; Pilot et al., 2018; vonHoldt et al., 2013). In India, human–wildlife interface is extensive and hence, it is crucial to monitor wild populations at these interfaces. The potential of systematic citizen science projects is undervalued and designing such large‐scale wildlife monitoring programs has received far less attention. Our results highlight the need to develop and standardize cost‐effective methods for hybrid detection using non‐invasive samples of canid species including dogs, wolves, and jackals, which coexist at the human‐wildlife interfaces.

Our results provide valuable information that can help understand wolf–dog interactions in India, and thereby, the conservation of wolf populations. Based on this report, we recommend systematic and periodic surveys of wolf–dog interactions that integrate conservation genetic approaches to better understand the dynamics of hybridization. Given the wolf's wide distribution range in India, and the relative rarity of observing such incidents, we suggest working with citizen scientists and wildlife enthusiasts to design and implement large‐scale, multi‐site conservation efforts. Our work here exemplifies a novel model for conservation in the future, involving citizens and cutting‐edge technology to acquire data at large spatial scales, and address conservation questions that would be important for ensuring the survival of imperiled species.

## AUTHOR CONTRIBUTIONS

**Abhinav Tyagi:** Conceptualization (equal); data curation (lead); formal analysis (lead); investigation (equal); methodology (lead); visualization (equal); writing – original draft (lead); writing – review and editing (equal). **Mihir Godbole:** Data curation (equal); writing – review and editing (equal). **Abi Tamim Vanak:** Conceptualization (equal); resources (equal); writing – review and editing (equal). **Uma Ramakrishnan:** Conceptualization (equal); funding acquisition (lead); project administration (lead); resources (lead); supervision (lead); writing – review and editing (equal).

## CONFLICT OF INTEREST STATEMENT

The authors declare no conflicts of interest.

## Supporting information


Data S1.

## Data Availability

The raw, demultiplexed ddRAD data generated in this study has been made available on NCBI Sequence Read Archive (SRA) database under BioProject number PRJNA962262.
