# Supplementary material for: Citizen science facilitates first ever genetic detection of wolf‐dog hybridization in Indian savannahs
Source: Ecol Evol. 2023 May 17;13(5):e10100. doi: 10.1002/ece3.10100 (PMC10191802; doi:10.1002/ece3.10100)
Supplement: Supplementary file 1 — Data S1. [file ECE3-13-e10100-s001.docx]

**Supplementary Materials**

**Table S1**: Details of all the samples used from NCBI to ascertain the genetic origin of suspected hybrid individuals.

| S.No. | Species | Sample Name | Location | Accession no. |
| --- | --- | --- | --- | --- |
| 1 | Village Dog | DogIND1 | India | SRR1061860 |
| 2 | Village Dog | DogIND2 | India | SRR1061958 |
| 3 | Village Dog | DogIND3 | India | SRR1061960 |
| 4 | Village Dog | DogIND4 | India | SRR1061964 |
| 5 | Village Dog | DogIND5 | India | SRR1061962 |
| 6 | Village Dog | DogNCHI1 | North China | SRR1061734 |
| 7 | Village Dog | DogNCHI2 | North China | SRR3384064 |
| 8 | Village Dog | DogSCHI1 | South China | SRR1061748 |
| 9 | Village Dog | DogSCHI2 | South China | SRR1061770 |
| 10 | Village Dog | DogUbk | Uzbekistan | SRR20326521 |
| 11 | Village Dog | DogNpl1 | Nepal | SRR20326527 |
| 12 | Village Dog | DogNpl2 | Nepal | SRR20326532 |
| 13 | Village Dog | DogIrn1 | Iran | SRR20326538 |
| 14 | Village Dog | DogIrn2 | Iran | SRR20326313 |
| 15 | Village Dog | DogKen1 | Kenya | SRR20326288 |
| 16 | Village Dog | DogKen2 | Kenya | SRR20326290 |
| 17 | Dhole | Dhole | Berlin zoo | SRR8049189 |
| 18 | Jackal | JackInd1 | India | SRR14777842 |
| 19 | Jackal | JackInd2 | India | SRR19863852 |
| 20 | Wolf | WolfInd1 | India | SRR14777843 |
| 21 | Wolf | WolfInd2 | India | SRR14777844 |
| 22 | Wolf | WolfInd3 | India | SRR14777845 |
| 23 | Wolf | WolfInd4 | India | SRR14777846 |
| 24 | Wolf | WolfInd5 | India | SRR13985171 |
| 25 | Wolf | WolfUS1 | North America | SRR8049197 |
| 26 | Wolf | WolfUS2 | North America | SRR8066602 |
| 27 | Wolf | WolfWA1 | West Asian | SRR8049193 |
| 28 | Wolf | WolfCA2 | Central Asian | SRR2827609 |
| 29 | Wolf | WolfEA2 | East Asian | SRR2827600 |
| 30 | Wolf | Wolf13ITL | Italy (Europe) | SRR1518519 |

*Two Indian dhole samples were obtained from Srivathsa et al., (2021), *Biological Conservation*, 256, 109028.

**Figure S1: (a)** Results of STRUCTURE analysis for K = 2, 3 and 4 using complete dataset of 35 individuals and 1573 SNPs. Each vertical line represents a unique individual. Dhole, Jackal and Indian wolves formed separate clusters at K = 4. These results are in congruence with Admixture results and PCA (Fig 2a & c). **(b)** Inference of optimal K value from STRUCTURE analysis ran for K = 1 to 6 by plotting of mean likelihood (ten replicates) at each value of K.

**
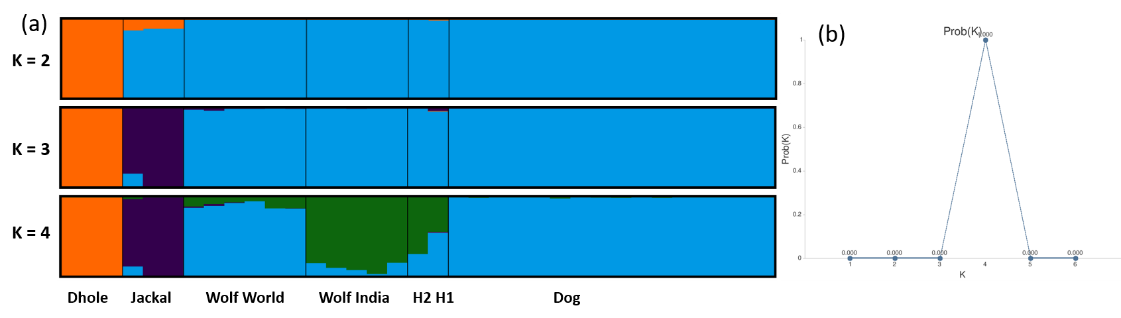
**

**Figure S2: (a)** Results of STRUCTURE analysis for K = 2 and 3 using only wolves and dogs along with both suspected hybrid individuals. Each vertical line represents a unique individual. Indian wolf individuals segregate from rest of the world wolves and dogs at K =2 and at K = 3, wolves segregates from dogs. H2 gets assigned with Indian wolf cluster, whereas H1 shows admixture between dog and Indian wolf. All dogs (q ≥ 0.80), Indian wolf (q ≥ 0.95) and rest of the world wolf (q ≥ 0.74) individuals along with H2 (q ≥ 0.85) were assigned to respective clusters with high probability. The assignment probability of H1 was found to be low (q ranges from 0.42-0.65). **(b)** Inference of optimal K value from STRUCTURE analysis ran for K = 1 to 6 by plotting of mean likelihood (ten replicates) at each value of K.

**
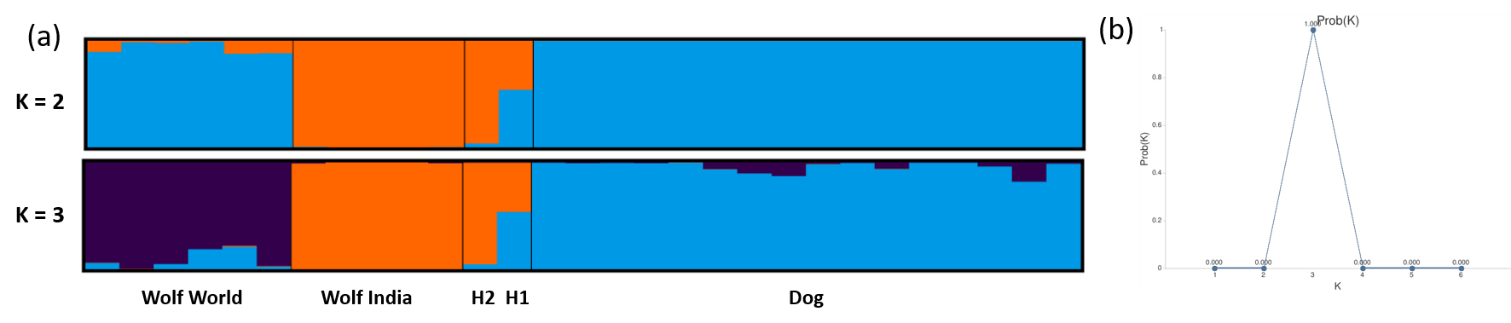
**
